# US Privacy Laws Go Against Public Preferences and Impede Public Health and Research: Survey Study

**DOI:** 10.2196/25266

**Published:** 2021-07-05

**Authors:** Cason Schmit, Theodoros Giannouchos, Mahin Ramezani, Qi Zheng, Michael A Morrisey, Hye-Chung Kum

**Affiliations:** 1 Population Informatics Lab Department of Health Policy and Management Texas A&M University College Station, TX United States; 2 Pharmacotherapy Outcomes Research Center College of Pharmacy University of Utah Salt Lake City, UT United States; 3 Department of Epidemiology and Biostatistics Texas A&M University College Station, TX United States

**Keywords:** privacy, law, medical informatics, conjoint analysis, surveys and questionnaires, public health, information dissemination, health policy, public policy, big data

## Abstract

**Background:**

Reaping the benefits from massive volumes of data collected in all sectors to improve population health, inform personalized medicine, and transform biomedical research requires the delicate balance between the benefits and risks of using individual-level data. There is a patchwork of US data protection laws that vary depending on the type of data, who is using it, and their intended purpose. Differences in these laws challenge big data projects using data from different sources. The decisions to permit or restrict data uses are determined by elected officials; therefore, constituent input is critical to finding the right balance between individual privacy and public benefits.

**Objective:**

This study explores the US public’s preferences for using identifiable data for different purposes without their consent.

**Methods:**

We measured data use preferences of a nationally representative sample of 504 US adults by conducting a web-based survey in February 2020. The survey used a choice-based conjoint analysis. We selected choice-based conjoint attributes and levels based on 5 US data protection laws (Health Insurance Portability and Accountability Act, Family Educational Rights and Privacy Act, Privacy Act of 1974, Federal Trade Commission Act, and the Common Rule). There were 72 different combinations of attribute levels, representing different data use scenarios. Participants were given 12 pairs of data use scenarios and were asked to choose the scenario they were the most comfortable with. We then simulated the population preferences by using the hierarchical Bayes regression model using the ChoiceModelR package in R.

**Results:**

Participants strongly preferred data reuse for public health and research than for profit-driven, marketing, or crime-detection activities. Participants also strongly preferred data use by universities or nonprofit organizations over data use by businesses and governments. Participants were fairly indifferent about the different types of data used (health, education, government, or economic data).

**Conclusions:**

Our results show a notable incongruence between public preferences and current US data protection laws. Our findings appear to show that the US public favors data uses promoting social benefits over those promoting individual or organizational interests. This study provides strong support for continued efforts to provide safe access to useful data sets for research and public health. Policy makers should consider more robust public health and research data use exceptions to align laws with public preferences. In addition, policy makers who revise laws to enable data use for research and public health should consider more comprehensive protection mechanisms, including transparent use of data and accountability.

## Introduction

Cleaning, integrating, and managing the uncertainty in chaotic real data is essential for reproducible science and to unleash the potential power of big data for biomedical research. This often requires access to very detailed data that inevitably raise privacy concerns. Despite the widespread use of personal information for big data purposes (eg, marketing, intelligence gathering, political campaigns), big data analytics are still challenged in health applications owing to concerns about privacy and complex and differing federal and state laws [1,2]. The patchwork of federal and state data protection laws poses a substantial challenge to leveraging data to promote health outcomes [1,2]. Data protection laws have 5 fundamental elements: (1) a definition of protected data, (2) definition of a regulated person or entity, (3) data use or disclosure restrictions, (4) data use or disclosure exceptions, and (5) penalties for violating the law. It is common for data protection laws to vary wildly in these 5 elements [2-6]. Consequently, it can be exceptionally difficult to understand which law (or laws) apply to a data project and whether a proposed data use is permitted. Often, the only commonality between different data protection laws is that they usually protect only identifiable data. Data that do not identify a person typically are not protected by US data protection laws. However, legal definitions for “identifiable” data or deidentification standards vary considerably [6]. This inconsistency encourages highly conservative measures to strip data of potential identifiers, which can severely limit data utility [6]. This reality poses a substantial barrier to cross-sectoral and cross-jurisdictional data uses relevant to health outcomes, including exploration of social determinants of health, retrospective database research studies, informatics research on decision support systems, digital ethology, and big data analytics in health (eg, precision public health) [2,7]. These barriers challenge efforts to rapidly leverage data in public health emergencies (eg, COVID-19).

An increasing number of published stories highlight the fact that different privacy protections apply in different contexts. For example, popular news stories have addressed how health information is treated differently when it is collected by health care providers as opposed to commercial companies such as Fitbit, Apple, or Ancestry.com [8,9]. Data use by health care providers is regulated by the Health Insurance Portability and Accountability Act of 1996 whereas data use by Fitbit or Apple is regulated by the Federal Trade Commission Act, which permits data use so long as they are neither unfair nor deceptive (ie, disclosed in a lengthy privacy policy) [10].

Recent high profile breaches (eg, Equifax) and scandals (eg, Facebook and the 2016 US election) have raised awareness of these different privacy standards [10,11]. Moreover, new data protection regulations in some jurisdictions have provoked debate and congressional inquiry into new federal privacy legislation [12-15]. Any new federal privacy law will necessarily address the 5 fundamental elements of data protection laws and will inevitably address the trade-off between privacy and utility [16,17]. Privacy risk in database studies is best minimized through a holistic approach that involves security technology (eg, encryption), data manipulation (eg, differential privacy), as well as good data governance models (eg, transparency) and legal protections. Legal protections can shield against a variety of harms. Alternatively, permitting certain data use can promote social benefits, including advancing knowledge and science, promoting public health, facilitating law enforcement, and enabling economic activity [18]. In the United States, the decision to permit or restrict certain data uses is determined by elected officials. These are policy choices with significant consequences for both individual interests (eg, privacy) and public benefit. Consequently, constituent input is critical to finding the right balance between individual privacy and public benefits.

The purpose of this paper is to report on the results of a nationally representative survey examining US residents’ preferences for which of their identifiable personal data should be available for use, by whom, and for what purposes. Prior research focusing on Americans’ attitudes on data use and privacy shows strong support for socially beneficial uses such as research [19-23]. However, few US privacy laws provide specific exceptions for data uses for research or public health [1,2]. Thus, information on how the US population views certain data uses in relation to other data uses is valuable, especially if one data use is currently restricted under US laws and the other is permitted. Such data would be extremely useful to US policy makers as they deliberate new data protection frameworks.

## Methods

### Study Design and Recruitment

In February 2020, we conducted a web-based survey to explore the comfort levels and the preferences of the US population when individually identifiable data is reused for different purposes without their consent. Potential participants were recruited via a third-party research company (Dynata) that specializes in deploying surveys by using nationally representative sampling. We sought to balance the sample on 6 targets based on population characteristics used by the census (gender, race/ethnicity, age, education, household income, and region) where possible. Our goal was to recruit 500 adult (≥18 years) US residents fluent in English to enable reasonable sample balancing [24]. To provide a demographic context for participants’ baseline privacy concerns, we included the validated Concern for Information Privacy instrument [25,26]. The Concern for Information Privacy instrument has 15 seven-point Likert scale questions (1=strongly disagree to 7=strongly agree) and provides a composite score with 4 subscales for privacy [25]. We estimated participants’ preferences on the potential reuse of their data with a choice-based conjoint analysis [27-30]. Choice-based conjoint analysis is built on the premise that an individual places different values on an option according to its characteristics and makes trade-off choices among alternatives based on the combination of characteristics. Conjoint analysis is a decomposition method because the implicit value for a characteristic is derived from some overall score for a profile consisting of 2 or more (conjoint) characteristics [27-29,31]. Choice-based conjoint analysis is commonly used in health care and economics research to understand clinical decision making, to assess patients’ preferences, evaluation, and willingness to accept new treatments and health states, and to promote shared decision making among patients and stakeholders by quantifying the relative importance that individuals place on different attributes and levels within those attributes [27-30,32-34].

We selected attributes based on 4 of the 5 elements of the data protection laws (excluding violation penalties) (Table 1). The “source of identifiable data” is related to the definition of protected data, “who” is related to the regulated entity, and the “proposed data use” is related to 2 different elements: legal restrictions and exceptions for data use or disclosure [2]. We selected attribute levels to correspond to various legal provisions permitting or restricting data reuse and to identify the nuances within the categories (eg, business vs nonprofit organization), which resulted in 80 different scenarios comprising different attribute levels (4×4×5) [10,35-38]. Of those, we excluded 8 as implausible or likely to confuse survey respondents (eg, government or nonprofit conducting profit-driven activity), leaving 72 different scenarios.

**Table 1:** Attributes and levels for data reuse scenarios.

Who	Purpose	Source of identifiable data
Researcher, University	Research, scientific knowledge dissemination	Education records
Nonprofit Organization	Promoting population health	Health records
Government	Identify criminal activity	Government program or activity
Business	Marketing, recruitment Profit-driven activity	Economic activity, customer behavior

**Figure 1:**
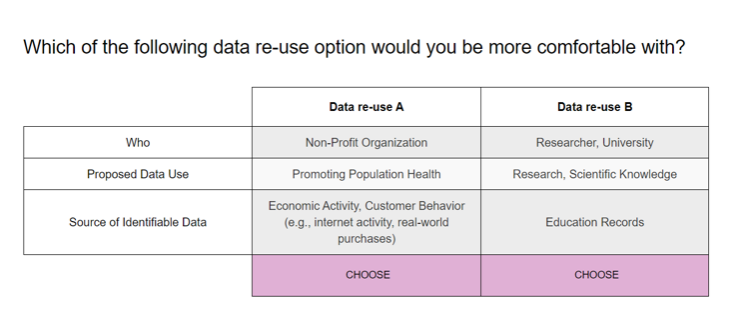
Sample pair scenario question.

Since it is not feasible and manageable to present all the possible combinations of each scenario to the participants, a fractional factorial design was used to randomly generate subsets of all the combinations, which were sufficient to obtain robust and meaningful differences in preferences through a standard web-based platform called "conjoint.ly", similar to that reported in previous work [33,34,39,40]. This resulted in 72 choice sets, with each set consisting of 12 pairs of data use scenarios that would allow for simulating participant preferences in the full space of data use scenario permutations. Each participant was randomly assigned to respond to one of the choice sets, and we asked each participant to select the data use scenario that they were the most comfortable with for each of their assigned 12 scenario pairs (Figure 1).

### Statistical Analysis

To estimate the parameters, we used a hierarchical Bayes regression model, and in estimating the parameters at the individual level, we generated 10,000 posterior draws by using the Markov chain Monte Carlo simulation [29,41,42]. This approach allowed the estimation of attributes and levels with the small amount of data collected from each respondent, while simultaneously accounting for the heterogeneity of preferences across and within individuals, the nested structure of the choices, and thus, the nonrandom preference variation of the respondents [29,34,41-43]. The value of 3 attributes was scaled to sum up to 100%, while the values of the levels within each attribute (part worth utilities) sum up to zero, with negative values indicating decreased and positive values indicating increased preferences. Finally, we used the sum of the estimated relative values (utilities) of different levels to identify and rank the alternative scenarios from the most to the least preferable. All measures were estimated at the individual level, which were then averaged and reported as the population mean with standard deviation in the results. Data analyses were conducted using the ChoiceModelR package in R [44]. This study was approved by our university institutional review board.

## Results

The survey was distributed to 687 individuals. Of them, 22 individuals declined to participate (3.2%), 157 did not fully complete the survey (22.8%), and 4 participant responses (0.6%) were marked as low quality based on detected participant behavior (eg, rapidly clicking through without mouse movement). This resulted in 504 respondents who fully completed the web-based survey (response rate 74.4%), which was our final analytic sample. Generally, we were able to meet our census sampling targets for gender, race/ethnicity, age, education, income, and census region (Table 2). In addition, although we did not try to balance for it, our sample’s health insurance coverage is similar to data published by the US Census Bureau [45]. Around half of the respondents had used a health care provider in the past year, around one-third had at least one chronic condition, and around 19.8% (100/504) of the respondents visited an emergency department in the past year. The overall privacy score was 5.8 (SD 1.1), which is consistent with the Concern for Information Privacy validation samples (ie, scores ranging from 5 to 6) [25].

**Table 2:** Sociodemographic data, clinical characteristics, and privacy attitude scores of the participants (N=504).

Participant characteristics			Values		Target sample percentage^a,b^
**Age categories (years), n (%)**					
	18-24	41 (8.1)		13.1	
	25-34	75 (14.9)		17.5	
	35-44	100 (19.8)		17.5	
	45-54	101 (20.0)		19.2	
	55-64	68 (13.5)		15.6	
	65 or older	89 (17.7)		17.2	
**Gender, n (%)**					
	Male	224 (44.4)		48.5	
	Female	278 (55.2)		50.5	
	Other/prefer not to answer	2 (0.4)		—^c^	
**Race categories, n (%)**					
	White	315 (62.5)		63.7	
	African American	77 (15.3)		12.2	
	Hispanic	51 (10.1)		16.4	
	Asian	46 (9.1)		4.7	
	Other	15 (3.0)		3.0	
**Income categories, n (%)**					
	$20,000 or less	103 (20.4)		19.9	
	$20,000 to $49,999	149 (29.6)		30.6	
	$50,000 to $99,999	137 (27.2)		29.1	
	$100,000 to $149,999	67 (13.3)		12.0	
	$150,000 or more	48 (9.5)		8.3	
**Educational level, n (%)**					
	High school or less	172 (34.1)		32.0	
	Some college completed	99 (19.6)		19.0	
	College degree	191 (37.9)		31.0	
	Master’s	37 (7.3)		—	
	PhD/doctoral	5 (1.0)		—	
**Region, n (%)**					
	Midwest	95 (18.8)		22.0	
	Northeast	126 (25.0)		18.2	
	South	174 (34.5)		36.2	
	West	109 (21.6)		23.6	
**Health insurance coverage, n (%)^b^**					
	Private	169 (33.5)		64.7	
	Medicare	112 (22.2)		17.7	
	Medicaid	83 (16.5)		17.9	
	Uninsured	52 (10.3)		8.5	
	VA/TRICARE	10 (2.0)		3.6	
	Multiple	78 (15.5)		14.5	
**Any chronic condition, n (%)**					
	No	319 (63.3)		—	
	Yes	181 (35.9)		—	
**Use of health care provider in the past year, n (%)**					
	No	93 (18.5)		—	
	Yes	256 (50.8)		—	
**At least one emergency department visit in the past year, n (%)**					
	No	404 (80.2)		—	
	Yes	100 (19.8)		—	
**Respondent is a primary care giver for someone else, n (%)**					
	No	423 (83.9)		—	
	Yes	77 (15.3)		—	
Concern for information privacy scores, mean (SD)			5.8 (1.1)		—

^a^Survey sampling targets based on census data.

^b^Insurance data were not used as the sampling target. These data show 2018 insurance statistics from the US census for survey sampling comparisons [45]. Our survey solicited mutually exclusive responses in contrast to the US census data, which do not exclude persons with multiple insurance types from these groups.

^c^Not available.

**Figure 2:**
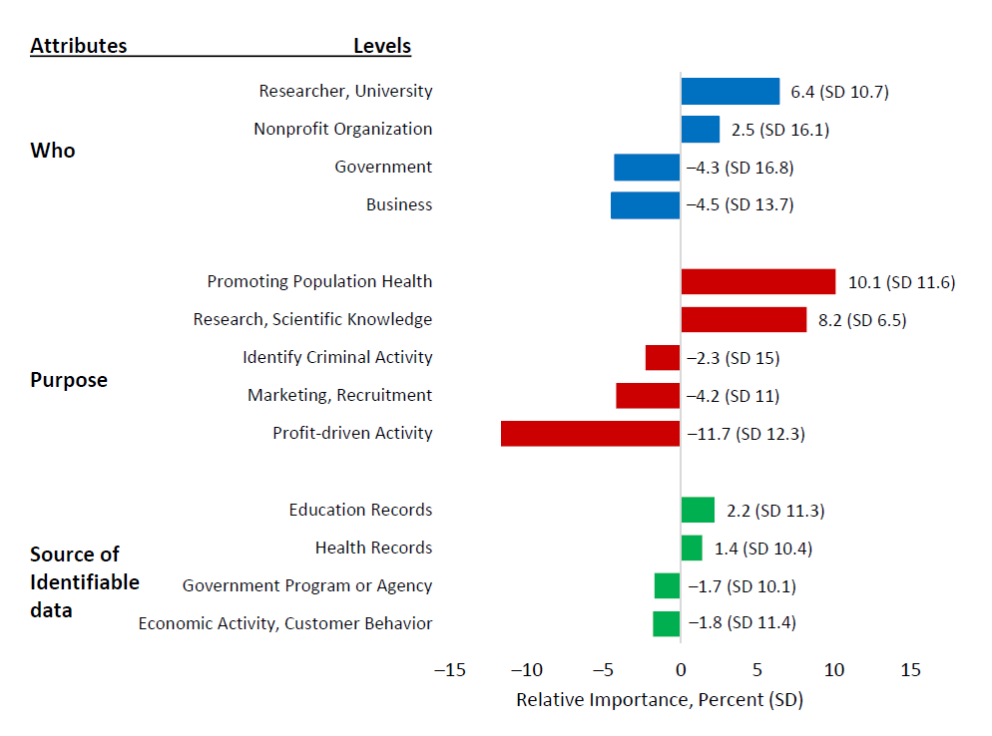
Relative importance by level within each attribute in percentage (SD).

Figure 2 presents the relative importance for the different levels within each attribute. Positive values indicate preference with higher values and reveal greater importance, while negative values indicate nonpreferred levels associated with potential data reuse. Participants were most comfortable with the reuse of identifiable data if the proposed data use was intended to promote population health (10.1%, SD 11.6) or promote science or research (8.2%, SD 6.5), if the data were used by university-affiliated researchers (6.4%, SD 10.7) or nonprofit organizations (2.5%, SD 16.1), and if the source of the data included educational (2.2%, SD 11.3) or health care records (1.4%, SD 10.4). In contrast, participants were least comfortable with data reuse by businesses (–4.5%, SD 13.7) or the government (–4.3%, SD 16.8) mainly for profit-driven (–11.7%, SD 12.3) or marketing (–4.2%, SD 11) activities based on governmental (–1.7%, SD 10.1) or economic activity data (–1.8%, SD 11.4). Overall, we observed higher differences in the values between the levels of the proposed data use attribute compared to other 2 attributes, particularly with the attribute related to the source of the identifiable data.

**Figure 3:**
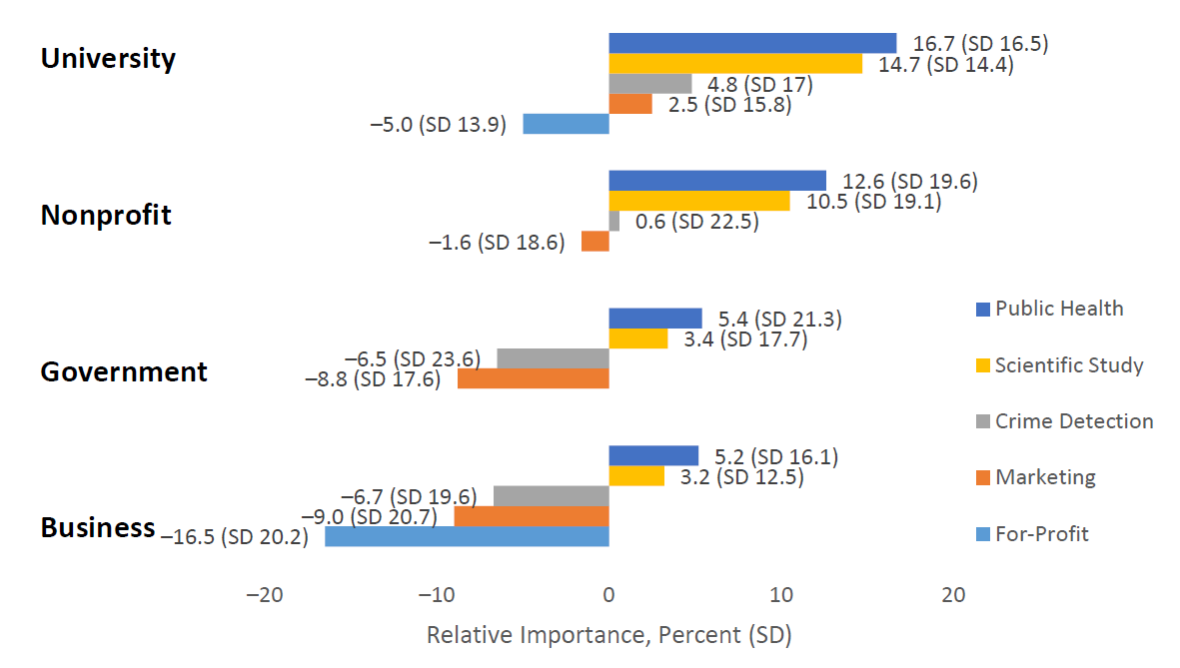
Public preferences for use of data by users and purpose in percentage (SD). Our survey did not pair “for-profit” purposes with government or nonprofit users because these pairings were implausible and likely to confuse survey respondents.

**Figure 4:**
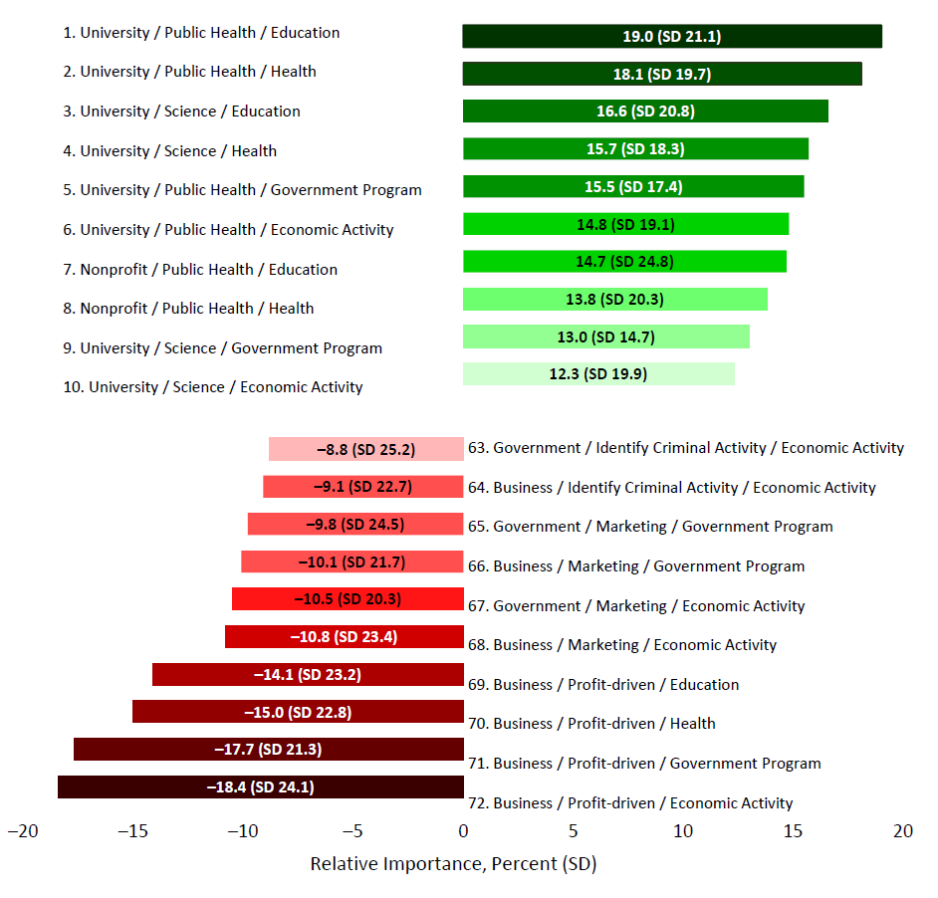
Top 10 and bottom 10 ranked data use scenarios derived from the sum of scenario attributes' relative values (who/use purpose/data source) in percentage (SD).

Figure 3 presents the relative importance of the different data use purposes by users. All data use activities were more preferred when conducted by universities or nonprofit organizations than when conducted by government or business. Public health and research activities received positive relative importance values regardless of who conducts the activity. Conversely, for-profit and marketing activities received near uniform negative relative importance values. Universities conducting marketing activities was the lone exception, which had a small but positive relative importance value (2.6%). The relative importance of identifying criminal activity was either positive (university and nonprofit) or negative (government and business) depending on the user. We also estimated the overall values that participants placed on each scenario. Figure 4 presents only the 10 highest and lowest ranked scenarios (Figure 4). The 4 lowest ranked scenarios all involved businesses using data for profit-driven purposes. The remainder of the lowest ranked scenarios involved business or government organizations engaging in marketing or identifying criminal activity. Eight of the 10 highest ranked scenarios involved universities/researchers engaging in scientific research or public health activities. Nonprofit organizations conducting population health programs represented the seventh and eighth highest ranked scenarios. We checked model validity by comparing the actual choices made by each participant with the estimated choices made for at least 90% of the last 50 iterations of the Markov chain Monte Carlo simulation. Precision (% of correct estimates) was good at 92% for the simulated model.

## Discussion

### Principal Findings

In contrast to federal and state laws, US residents make little distinctions across types of data. However, they express much more favorable preferences for uses by academic researchers and nonprofit organizations than by the government or the business community. Moreover, all types of users consistently preferred uses that focus on public health and scientific research rather than on crime detection, marketing, or for-profit activities. Our data demonstrate interesting inconsistencies between public preferences and US privacy laws. These inconsistencies are best exemplified by our participants’ most preferred data reuse (researchers using education data to promote population health) and least preferred data reuse (businesses using consumer data for profit). Ironically, our data indicate that the US public’s most preferred data reuse scenario is currently prohibited under the federal Family Educational Rights and Privacy Act of 1974 while the US public’s least preferred data reuse is completely legal and ubiquitous under the permissive Federal Trade Commission Act [38,46]. A recent review of 22 federal data protection frameworks funded by the Network for Public Health Law indicates that few data protection laws have a general research exception and fewer have a specific exception for public health uses. However, these data uses were by far the most preferred options of those we presented to our participant and were consistently preferred, regardless of who was the data user. Yet, public health uses are treated differently under different US laws [6]. For example, the law protecting substance use treatment records has hamstrung the use of data in the present opioid epidemic response, while the laws covering cell phone location data have permitted public health officials to track entire populations in the current COVID-19 pandemic [47,48].

Our participants also strongly favored data uses by universities and nonprofit organizations. Both universities and nonprofit organizations received higher preference ratings for all data use activities when compared to those received by the government or businesses. In some cases, activities that participants viewed as heavily undesirable when conducted by the government or business (crime detection, marketing) were rated favorably when conducted by a nonprofit organization or university. In contrast, the least preferred scenarios involved data reused for profit-driven or marketing activities by businesses or government. Mistrust in government has been documented in other research on attitudes of research and is perhaps unsurprising in the present partisan political environment [21]. Negative preference ratings for businesses, profit-driven activities, and marketing are likely due to frequent stories of controversial data use, mismanagement, or breaches that are all too common in the news [49]. This finding is consistent with other research documenting strong public attitudes in favor of altruistic goals and skepticism of data uses that advance specific individual or private (ie, for-profit) interests [19,21].

We did find some preference differences for certain data types, but these differences were modest. Our data show that the public prefers the use of health or educational data (both heavily regulated under US laws) as compared to government data or economic data. Still, our data do not show any strong preferences. The public seems to view data as data. We noted that 4 of the 5 data use purposes we included in our study fall neatly into 2 broad categories: altruistic purposes and self-serving purposes. Public health and scientific purposes both ultimately contribute to the greater good, and our data suggest that these purposes are strongly preferred by the US public, regardless of who is doing the activity. In contrast, our respondents generally found those activities that are primarily self-serving (ie, profit-driven or marketing/recruitment activities) undesirable, regardless of who was doing the activity. The lone exception was marketing by universities, which received a modest positive relative importance score. Consequently, it could be that our participants based some of their preference decisions on whether they saw the data use as contributing to an altruistic or common good objective as opposed to primarily benefiting the data user’s self-interests.

Identifying criminal activity was the one data use that does not neatly fit in the broad categories of altruistic or self-serving purposes. While law enforcement clearly has some social benefits (as do all the activities used in our study), identifying criminal activity implies punishment for some individuals. Consequently, it is not entirely altruistic and not entirely self-serving. Interestingly, participant preferences for identifying criminal activity seemed to vary depending on the data user. Universities and nonprofit organizations both received positive relative importance scores whereas governments and businesses received negative scores. Just as with other data uses, it could be that participants positively associate universities and nonprofit organizations with motivations more in line with social benefits rather than individual benefits.

Collectively, our results do not support the current patchwork of US data protection laws. Many US data protection laws focus primarily on the type of data (ie, health, education, governmental program data), but our respondents were fairly indifferent toward these distinctions. Instead, our findings suggest that the US public is much more interested in who is using the data and for what purposes the data are being used. In particular, our results suggest that the US public has a strong preference for data uses that promote the common good as opposed to individual or self-serving interests.

In fact, findings suggest that US preferences more closely align with a comprehensive data protection framework such as the General Data Protection Regulation enacted by the European Union where rules vary based on data use but are broadly applicable to all identifiable data [50]. For example, the General Data Protection Regulation has broad applications and express provisions permitting scientific research and activities in the public interest (eg, public health) [51,52]. Policy makers who revise laws to increase access to data for research and public health can support data protection through new security standards. A 2009 report by the Institute of Medicine, “Beyond the HIPAA Privacy Rule: Enhancing Privacy, Improving Health through Research” [53] argued for a different data protection approach to “enhance privacy protections through improved data security, increased transparency of activities and policies, and greater accountability.” These good governance practices, as opposed to strict prohibitions on uses and disclosures (ie, for research or public health), provide a way to protect individuals while permitting big data applications (eg, linking data from different sources) with social benefits. These results provide strong public support for continued efforts to make data available for research and public health.

### Limitations

There are 2 important limitations. We did not capture the universe of data use possibilities; therefore, the measured participants’ preferences are relative to the 72 provided scenarios. Additionally, this design measured participants’ preferences rather than acceptability, meaning that a participants’ least preferred scenario could still be acceptable to them or the most preferred scenario might be unacceptable.

### Conclusion

Importantly, these results support a close re-examination of the absence of public health and research data use exceptions in US laws. It is clear that the US public strongly prefers using data to promote population health (as compared to other legal data uses); yet, few laws allow this kind of exception. The Family Educational Rights and Privacy Act provides an excellent example, given that it does not have a public health exception (or a research exception that permits exploring health implications) despite being one of the most potent known social determinants of health. Moreover, the absence of these data use exceptions within the current patchwork of inconsistent US data protection laws persistently frustrates secondary database researchers and public health professionals, thereby delaying, impeding, or increasing the cost of data-intensive scientific discovery and public health practice [1,2,4,6]. These findings clearly show that there is poor alignment between the present US legal data protection framework and the preferences of the US population.
